# Glycomacropeptide Prevents Iron/Ascorbate-Induced Oxidative Stress, Inflammation and Insulin Sensitivity with an Impact on Lipoprotein Production in Intestinal Caco-2/15 Cells

**DOI:** 10.3390/nu12041175

**Published:** 2020-04-22

**Authors:** Mathilde Foisy-Sauvé, Lena Ahmarani, Edgard Delvin, Alain T. Sané, Schohraya Spahis, Emile Levy

**Affiliations:** 1 Research Centre, CHU Ste-Justine, Montreal, QC H3T 1C5, Canada; mfois067@uottawa.ca (M.F.-S.); lena_ahm@yahoo.com (L.A.); delvine@sympatico.ca (E.D.); sanealaintheo@gmail.com (A.T.S.); schohraya.spahis@recherche-ste-justine.qc.ca (S.S.); 2 Department of Nutrition, Université de Montréal, Montreal, QC H3T 1A8, Canada

**Keywords:** glycomacropeptide, oxidative stress, inflammation, insulin sensitivity, metabolic syndrome, milk-derived bioactive peptide

## Abstract

Background. Metabolic Syndrome (MetS), a major worldwide concern for the public health system, refers to a cluster of key metabolic components, and represents a risk factor for diabetes and cardiovascular diseases. As oxidative stress (OxS) and inflammation are the major triggers of insulin sensitivity (IS), a cardinal MetS feature, the principal aim of the present work is to determine whether glycomacropeptide (GMP), a milk-derived bioactive peptide, exerts beneficial effects on their expression. Methods. Fully differentiated intestinal Caco-2/15 cells are used to evaluate the preventive action of 2 mg/mL GMP against OxS and inflammation induced by the mixture iron-ascorbate (Fe/Asc) (200 μM:2 mM). The potency of GMP of decreasing the production of lipoproteins, including chylomicrons (CM), very-low-density lipoproteins (VLDL) and low-density lipoproteins (LDL) is also assessed. Results. The administration of GMP significantly reduces malondialdehyde, a biomarker of lipid peroxidation, and raises superoxide dismutase 2 and glutathione peroxidase via the induction of the nuclear factor erythroid 2–related factor 2, a transcription factor, which orchestrates cellular antioxidant defenses. Similarly, GMP markedly lowers the inflammatory agents tumor necrosis factor-α and cyclooxygenase-2 via abrogation of the nuclear transcription factor-kB. Moreover, GMP-treated cells show a down-regulation of Fe/Asc-induced mitogen activated protein kinase pathway, suggesting greater IS. Finally, GMP decreases the production of CM, VLDL, and LDL. Conclusions. Our results highlight the effectiveness of GMP in attenuating OxS, inflammation and lipoprotein biogenesis, as well as improving IS, the key components of MetS. Further investigation is needed to elucidate the mechanisms mediating the preventive action of GMP.

## 1. Introduction

The intestine plays an essential role in nutrient digestion and absorption. It also actively participates in host protection and metabolism by housing much of the human microbiota [1,2]. It exerts a marked influence on health, being equipped with a strong neuroendocrine system, producing a number of centrally and peripherally metabolically effective peptides [3,4,5]. In addition, not only does the intestine act as a physical barrier to protect the body from the harsh luminal environment, but also through its intraluminal microbiota in order to modulate energy metabolism and inflammation processes associated with obesity and diabetes [6].

The assertion that the gastrointestinal (GI) tract represents a meaningful target to treat conditions conducive to cardiometabolic disorders (CMD) is of clinical relevance. For instance, Schauer et al. [7] have shown in a 5-year prospective randomized controlled trial that bariatric surgery coupled to intensive medical treatment results in a better control of dysglycemia and remission of type 2-diabetes than medical treatment alone. However, when bariatric surgery does not represent an optimal option, uncovering new nutrients with a significantly intestinal metabolic impact is crucial to assure prevention and treatment of CMD. In this context, there is a growing interest in bioactive peptides, such as growth and immunity factors, derived from human and bovine milk given their high potential in assuring health benefits for neonates, infants, adolescents and adults [8,9,10,11,12,13]. However, most of the beneficial effects described for milk-derived peptides have not been exhaustively investigated in link with the GI system. Furthermore, little is known about their effects on CMD and even less on the related mechanistic aspects.

Bovine kappa-caseino glycomacropeptide or glycomacropeptide (GMP), a 64-amino acid glycophosphopeptide, has been described as one of the most biologically active milk component [14,15]. This carbohydrate-containing compound can survive gastric transit without hydrolysis and is absorbed intact into the bloodstream [16,17]. Even if 30 years ago GMP was considered as a source of energy, it now becomes obvious that it has also the potential of modulating various biological processes, including binding of cholera toxin and *E. Coli* enterotoxins, inhibition of bacterial and viral adhesion, suppression of gastric secretion, and promotion of bifidobacterial growth [18,19,20,21,22]. The anti-inflammatory and immunomodulatory effects of GMP have been reported in rodent models of inflammatory bowel disease [23,24], murine spleen and bone marrow dendritic cells [25,26], as well as in lymphocytes and macrophages [27]. Until very recently, few animal studies have enlightened the capacity of GMP to attenuate metabolic disorders such as diabetes and dyslipidemia without addressing the underlying mechanisms [28,29,30]. Lately, Yuan et al. [30] demonstrated that supplementing diabetic mice with a GMP hydrolysate significantly reduced fasting blood glucose, restored insulin production, improved insulin resistance (IR), increased skeletal muscle glycogen content, and reduced systemic inflammation while modifying the gut microbiota.

To our knowledge, there is very little research focusing on the impact of GMP on oxidative stress (OxS) and inflammation pathways in enterocytes. Therefore, the major aim of this study is to determine whether GMP modulates OxS and inflammation, two central processes favoring IR using the well-established human-derived immortalized Caco-2/15 cell line. These intestinal cells spontaneously differentiate into polarized mature enterocytes under standard culture conditions and lend themselves to the in vitro study of human gut in view of its efficient intestinal transport processes [31].

## 2. Materials and Methods

### 2.1. Caco-2/15 Cell Culture

The human epithelial colorectal adenocarcinoma Caco-2/15 cell line, a stable clone of the parent Caco-2 cells (ATCC^®^ HTB-37^™^, American Type Culture Collection, Rockville, MD, USA), was obtained from Dr. JF Beaulieu (Department of Cellular Biology, Faculty of Medicine, Université de Sherbrooke, Sherbrooke, QC, Canada) [32,33]. Caco-2/15 cells were grown at 37 °C with 5% CO_2_ in minimal essential medium (MEM) (GIBCO-BRL, Grand Island, NE, USA) containing 1% penicillin-streptomycin and 1% MEM nonessential amino acids (GIBCO BRL) and supplemented with 10% decomplemented fetal bovine serum (FBS) (Flow, McLean, VA, USA) as described previously [34]. Caco-2/15 cells were maintained in T-75-cm^2^ flasks (Corning Glass Works, Corning, NY, USA). Cultures were split (1:5) when they reached 70–90% confluence, by use of 0.05% trypsin-0.5 mM EDTA (GIBCO-BRL). For individual experiments, cells were plated at a density of 1 × 10^6^ cells/well on 6-well polycarbonate plates (Costar, Cambridge, MA, USA). Specifically, for lipoprotein assessment, cells were plated on 24.5-mm polycarbonate Transwell filter inserts with 0.4-µm pores (Costar), in MEM (as described above) supplemented with 5% FBS. The inserts were placed into six-well culture plates, permitting separate access to the apical and basolateral compartments of the monolayers. Cells were cultured for fourteen days at which point the Caco-2/15 cells are highly differentiated into polarized mature enterocytes and appropriate for lipid metabolism [33,34]. The medium was refreshed every other day.

### 2.2. Caco 2/15 Cell Integrity

Viability, morphology, and differentiation assays were performed to estimate Caco 2/15 cell integrity. Cell viability was evaluated with 3-(4,5-dimethyldiazol-2-yl)-2,5 diphenyl Tetrazolium Bromid (MTT, Sigma) after pre-treatment with different doses (0.5, 1, and 2 mg/mL) of purified GMP powder and incubation with 200 μM iron (II) sulphate heptahydrate (Fe, Sigma-Aldrich, St Louis, MO, USA) and 2 mM ascorbate (Asc, Sigma-Aldrich, St Louis, MO, USA) complex +/− GMP for 6 h at 37 °C [33,34,35]. Following cell incubation, the medium was aspirated and replaced with an MTT solution (0.5 mg/mL). Incubation of cells for 2 h at 37 °C with 5% CO_2_ enables MTT oxidation by the succinate dehydrogenase enzyme in viable cells. The MTT solution was then aspirated and 500 μL of dimethyl sulfoxide was added to each well to dissolve the resulting blue formazan crystals. The absorbance was measured at 535 nm with DMSO as a blank. Since 2 mg/mL of GMP caused no toxicity, this concentration was selected for the subsequent experiments. To estimate cellular differentiation and barrier integrity, villin, and occludin protein expression were assessed. Finally, monolayer intactness and physical barrier function were tested by evaluating morphology and transepithelial electric resistance [34].

### 2.3. Induction of Oxidative Stress and Inflammation

Fourteen days post confluence, the cells were washed twice with PBS and incubated in serum-free Eagle’s minimal essential medium (EMEM) supplemented with 1% penicillin-streptomycin and 1% non-essential amino acid (NEAA) for 18 h in the presence or absence of GMP. Then after, the medium was removed, and cells were cultured with Fe/Asc in serum free EMEM supplemented with 1% penicillin-streptomycin and 1% NEAA (pH 7.4) +/− GMP for 6 h at 37 °C to induce OxS and inflammation. This strong oxygen radical-generating system was employed to challenge cells and to evaluate their capacity to respond to an external pro-oxidant and pro-inflammatory stimulus as described previously [33,34,35].

### 2.4. Insulin Pathways

Following the same protocol above, two hours before the end of the six-hour incubation with Fe/Asc, insulin (Humulin R) was added at a final concentration of 100 nM to evaluate the effect of the OxS on insulin sensitivity (IS) arising from signalization pathways [33].

### 2.5. Malondiadehyde Measurement

Estimation of lipid peroxidation was assessed by malondialdehyde (MDA) using a fluorescence detector HPLC as described previously [36,37,38,39,40,41].

### 2.6. Protein Expression Analysis by Immunoblotting

Following incubation with various treatments, Caco-2/15 cells were lysed in ice-cold buffer containing 20 mM Tris-HCl (pH 7.4), 150 mM NaCl, 1 mM Na2EDTA, 1 mM EGTA, 1% NP-40, 1% deoxycholate, 2.5 mM sodium pyrophosphate, 1 mM Na2VO4, 1 μg/mL leupeptin, and 1 mM PMSF. Protein concentration of each sample was determined using the Bradford method (Bio-Rad). Proteins were then denatured for 5 min in sample buffer containing SDS and ß-mercaptoethanol. Homogenates containing 15 μg total proteins were separated on a 10% SDS-polyacrylamide gel according to protein molecular weights and were electroblotted onto nitrocellulose membranes. Fat-free milk was used to block nonspecific sites of the membranes before adding primary antibodies overnight at 4 °C: villin (94 kDa, 1/1000, BD Biosciences); occludin (59 kDa, 1/1000, Abcam); tumor necrosis factor-alpha (TNF-α, 26 kDa, 1/1000, ThermoFisher scientific, Waltham, MA, USA); cyclooxygenase-2 (COX-2, 70 kDa, 1/1000, Novus); nuclear factor kappa-B (NF-κB, 65 kDa, 1/5000, Santa Cruz Biotechnology); nuclear factor erythroid-2-related factor 2 (NRF2, 110 kDa, 1/1000, Abcam); inhibitor of kappa B (IκB, 39 kDa, 1/1000, Cell Signaling Biotechnology); glutathione peroxidase 1 (GPx1, 26 kDa, 1/1000, Novus Biologicals); superoxide dismutase 2 (SOD2, 21 kDa, 1/3000, Invitrogen); phospho-Akt (p-Akt^-Ser473^, 60 kDa, 1/1000, ThermoFisher scientific); AKT (60 kDa, 1/1000, Cell signaling); p38 mitogen activated protein kinase (p38MAPK, 43 kDa, 1/1000, ThermoFisher scientific); phospho-p38 MAPK (43 kDa, 1/1000, Cell signaling); PI3 Kinase (PI3K, 85 kDa, 1/3000, Invitrogen); phospho PI3K p85 (85 kDA, 1/500, Abcam); AMP-activated protein kinase alpha (AMPKα and phospho AMPKα^Thr172^, 62 kDa, 1/1000, Cell signaling) and β-actin (43 kDa; 1/10 00, Sigma-Aldrich), as a housekeeping protein. Reactive bands were captured using a ChemiDoc MP Imaging System (Bio-Rad). All data are expressed as the ratio of target protein to β-actin in the same sample.

### 2.7. Lipoprotein Assessment

Cells were seeded at a density of 1 × 10^6^/well in six-well polycarbonate Transwell filter inserts plates (Costar Cambridge, MA, USA) containing 2.5 mL of culture medium in the basolateral compartment and 1.5 mL in the apical one. The culture medium was refreshed every 3 days. Fourteen days after seeding, cells were washed twice with PBS and supplied with 2.5 mL serum-free EMEM in the basolateral chamber, and 1.5 mL serum-free EMEM containing (BSA/oleic acid) (1:5, *v*:*v*, pH 7.4) in the apical chamber. GMP (2 mg/mL) was added to determine its effects on lipoprotein production. After 18 h, the culture medium was discarded and replaced with fresh 2.5 mL serum-free EMEM and 1.5 mL radiolabeled [^14^C]-oleic acid (0.45 μCi) (53 mCi/mmol; Amersham, Oakville, ON, Canada) ± GMP (2 mg/mL) in the basolateral and the apical compartments, respectively. After a 24 h-incubation, media were collected from the two chambers and cells were washed twice with cold PBS and scraped in 1 mL of lysis buffer (1× TBS, pH 7.4; 5 mM EDTA; 0.1% SDS; 1% Triton ×100; 0.5% Sodium deoxycholate) containing 10 μL/mL of each anti-protease (PMSF, Leupeptin, and Pepstatin).

### 2.8. Isolation of Lipoproteins

Newly synthesized lipoproteins were separated from the basolateral medium supplemented with anti-proteases and plasma lipid carrier (4:1, *v*/*v*) to efficiently isolate *de novo* produced lipoproteins. Isolation was performed by sequential ultracentrifugation using a TL-100 ultracentrifuge as per our usual technique [33,34,35,38,42,43,44].

### 2.9. Statistical Analysis

All values are expressed as the mean ± SEM of at least 3 different experiments carried out in triplicate. Data were analyzed by one-way ANOVA followed by the Tukey’s multiple comparisons test using PRISM 7.0 (GraphPad Software, San Diego, CA, USA). Differences were considered significant at *p* < 0.05.

## 3. Results

### 3.1. Caco-215 Cell Integrity

**Figure 1:**
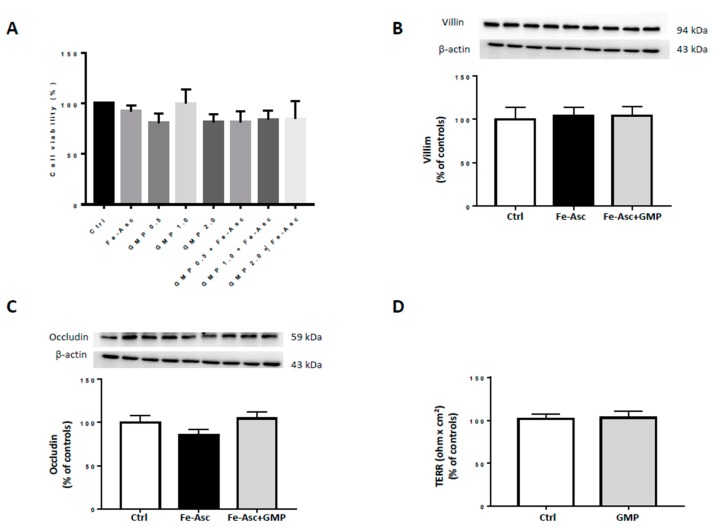
Effects of treatments on cell integrity in Caco-2/15 cells. Integrity of the cell monolayer was evaluated by cell viability, differentiation, and tight junction assays using fully differentiated Caco-2/15 cells. Different doses (0.5, 1, and 2 mg/mL) of purified glycomacropeptide (GMP) powder were added to Caco-2/15 cells 18 h before incubation with Fe/Asc (200 µM/2 mM) for 6 h at 37 °C for the (**A**) MTT assay. A dose of 2 mg/mL was selected for the assessment of (**B**) villin and (**C**) occludin protein expression as well as (**D**) transepithelial electrical resistance (TEER). Results represent the means ± SEM of *n* = 3 independent experiments in triplicate.

To ensure the consistency of our experiments, we first examined the integrity of Caco-2/15 following the addition of Fe/Asc and GMP. Compared to controls, no significant alterations were noted in cell viability using trypan blue staining and MTT assay, as described previously [34] after 24h-incubation (Figure 1A). We tested different doses (0.5, 1, and 2 mg/mL) for the MTT assay and 2 mg/mL was used for subsequent experiments as it did not alter cell viability and exerted the most beneficial effects on OxS (data not shown). Similarly, the barrier integrity and differentiation of Caco-2/15 cells were not modified as observed by the transepithelial electrical resistance, occludin and villin protein expression, respectively (Figure 1B–D). These results indicate that the administration of Fe/Asc and GMP do not exert cytotoxic effects on intestinal Caco-2/15 cells.

### 3.2. Effects of Glycomacropeptide (GMP) on Lipid Peroxidation and Oxidative Stress (OxS)

**Figure 2:**
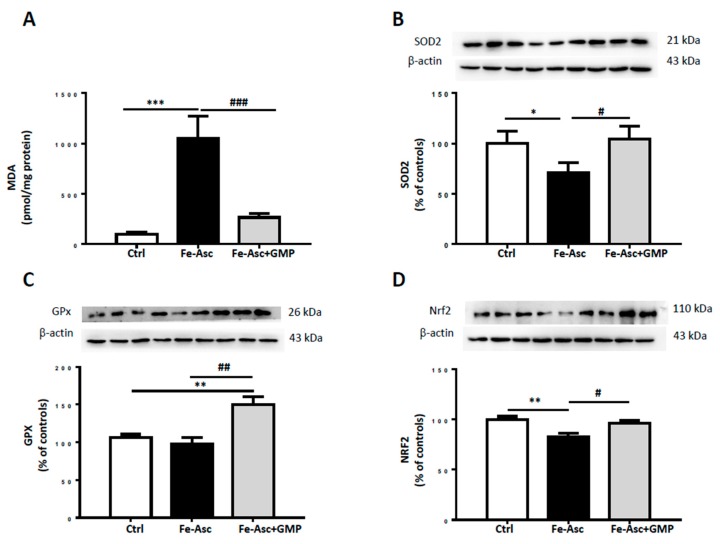
Effects of GMP on lipid peroxidation and antioxidant defense in Caco-2/15 cells. Caco-2/15 cells were pre-incubated for 18 h before treatment with Fe/Asc (200 µM/2 mM) +/− GMP (2 mg/mL) for 6 h at 37 °C as described in Materials and Methods. (**A**) Levels of malondialdehyde (MDA), a biomarker of lipid peroxidation, were measured in cell lysates. Protein expression of the anti-oxidative markers (**B**) superoxide dismutase 2 (SOD2), (**C**) glutathione peroxidase (GPx), and (**D**) nuclear factor erythroid 2–related factor 2 (Nrf2) was determined by Western blot. Results represent the means ± SEM of *n* = 3 independent experiments in triplicate. * *p* < 0.05, ** *p* < 0.01, *** *p* < 0.001 vs. controls (Ctrl). ^#^ *p* < 0.05, ^##^ *p* < 0.01, ^###^ *p* < 0.001 vs. Fe/Asc-treated cells.

The extent of lipid peroxidation following the treatment of Caco-2/15 cells with Fe/Asc (200 µM, 2 mM) during 6h was assessed to determine the malondialdehyde (MDA) levels [33]. HPLC analyses indicated a 10-fold increase in MDA following the administration of the oxygen free radical generating system Fe/Asc as compared with controls, and treatment with GMP strongly counteracted Fe/Asc-mediated lipid peroxidation (Figure 2A).

As failure of antioxidant defense may favour the induction of OxS, we examined the important endogenous antioxidant enzymes (SOD2, GPx). Treatment of Caco-2/15 cells with Fe/Asc caused a significant decrease in SOD2 protein expression with a slight drop of GPx. However, incubation with GMP blunted the Fe/Asc-mediated fall of SOD2 and upregulated GPx (Figure 2B,C), as well as restored NRF2, a critical transcription factor involved in cellular defense mechanisms against OxS (Figure 2D).

### 3.3. Effects of GMP on Inflammation

**Figure 3:**
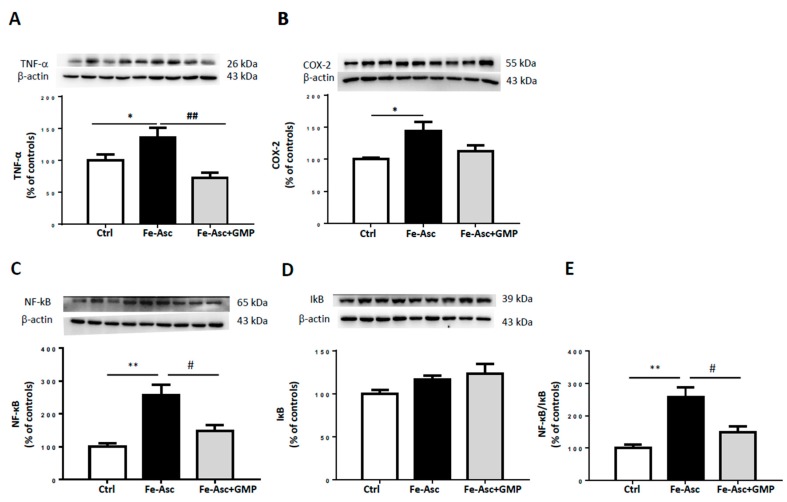
Effects of GMP on inflammation in Caco-2/15 cells. Caco-2/15 cells were pre-incubated for 18 h before treatment with Fe/Asc (200 µM/2 mM) +/− GMP (2 mg/mL) for 6 h at 37°C as described in Materials and Methods. Protein expression of the inflammatory biomarkers (**A**) Tumor necrosis factor alpha (TNF-α), (**B**) Cyclooxygenase-2 (COX-2), (**C**) Nuclear transcription factor-kappa B (NF-κB), and (**D**) Inhibitor of kappa B (IκB) was determined by Western blot. (**E**) The NF-κB/IκB ratio was calculated. Results represent the means ± SEM of *n* = 3 independent experiments in triplicate. * *p* < 0.05, ** *p* < 0.01 vs. controls (Ctrl). ^#^ *p* < 0.05, ^##^ *p* <0.01 vs Fe/Asc-treated cells.

The protein expression of TNF-α, a powerful inflammatory mediator, was enhanced following the incubation of Caco-2/15 cells with Fe/Asc (Figure 3A). However, GMP-treated Caco-2/15 cells reduced TNF-α levels. As COX-2 amplifies and perpetuates the inflammatory condition by producing eicosanoids, its expression was also estimated by Western blot analysis. Our experiments showed that Fe/Asc elicited elevated COX-2 protein expression, which was dulled by GMP (Figure 3B). Moreover, Caco-2/15 cells exposed to Fe/Asc displayed a high protein expression of NF-κB as well as NF-κB/IκB ratio, which is indicative of an elevated transcriptional activation of its target inflammatory genes. GMP was found to blunt the activation of the NF-κB pathway in response to Fe/Asc (Figure 3C–E).

### 3.4. Effects of GMP on Insulin Sensitivity Deriving from Insulin Signaling Pathways

**Figure 4:**
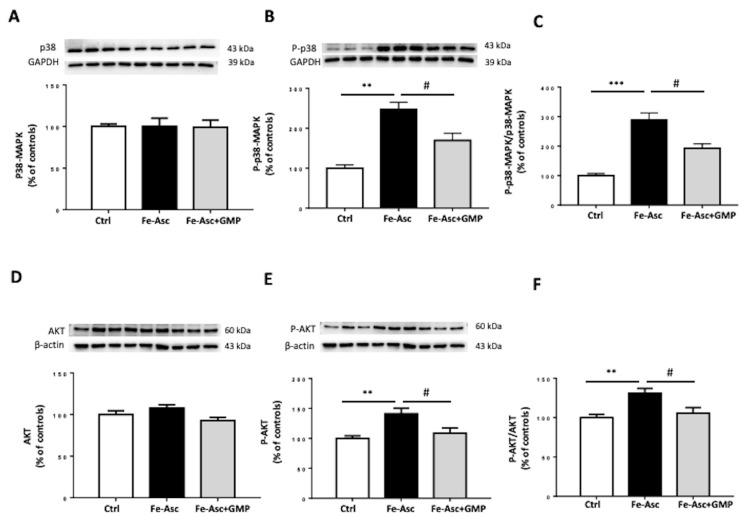
Effects of GMP on insulin sensitivity in Caco-2/15 cells. Caco-2/15 cells were pre-incubated for 18 h before treatment with Fe/Asc (200 µM/2 mM) +/− GMP (2 mg/mL) for 6 h at 37 °C. Two hours before the end of the six hour-incubation with Fe/Asc, insulin (100 nM) was added to evaluate insulin sensitivity as described in Materials and Methods. Protein expression of (**A**) p38-MAPK, (**B**) phospho-p38-MAPK, (**D**) AKT and (**E**) phospho-Akt was determined by Western blot. (**C**) Phospho p38-MAP/p38-MAPK and (**F**) phospho-Akt/AKT ratios were calculated. Results represent the means ± SEM of *n* = 3 independent experiments in triplicate. ** *p* < 0.01, *** *p* < 0.001 vs. Controls (Ctr); ^#^ *p* < 0.05 vs. Fe/Asc Caco-2 cells treated with insulin.

**Figure 5:**
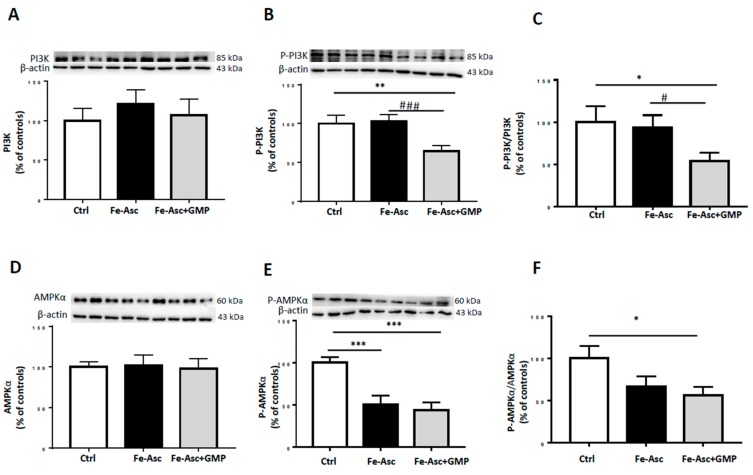
Effects of GMP on insulin signaling in Caco-2/15 cells. Caco-2/15 cells were pre-incubated for 18 h before treatment with Fe/Asc (200 µM/2 mM) +/− GMP (2 mg/mL) for 6 h at 37 °C. Two hours before the end of the six hours incubation with Fe/Asc, insulin (100 nM) was added to evaluate insulin signaling as described in Materials and Methods. Protein expression of (**A**) PI3K, (**B**) phospho-PI3K, (**D**) AMPKα and (**E**) phospho-AMPKα was determined by Western blot. (**C**) Phospho-PI3K/PI3K and (**F**) phospho-AMPKα/AMPKα ratios were calculated. Results represent the means ± SEM of *n* = 3 independent experiments in triplicate. *p < 0.05, ** *p* < 0.01, *** *p* < 0.001 vs. controls (Ctrl). ^#^ *p* < 0.05, ^###^ *p* < 0.001 vs. Fe/Asc Caco-2 cells treated with insulin.

To investigate the impact of GMP on IS, the two main insulin signaling pathways were considered: the MAPKs and PI3K/AKT pathways [45,46]. Caco-2/15 cells were treated with insulin. Figure 4 shows that protein levels of phosphorylated forms of p38-MAPK and AKT were increased following Fe/Asc incubation whereas GMP displayed potency to prevent their activation (Figure 4B,E). A similar trend was noticed when the ratio was calculated (Figure 4C,F). However, insulin treatment caused no changes in total PI3K and AMPK protein expression (Figure 5A,D), but GMP downregulated PI3K and AMPK phosphorylation (Figure 5B,E) as well as the calculated ratio (Figure 5C,F).

### 3.5. Influence of GMP on Lipoprotein Production

**Figure 6:**
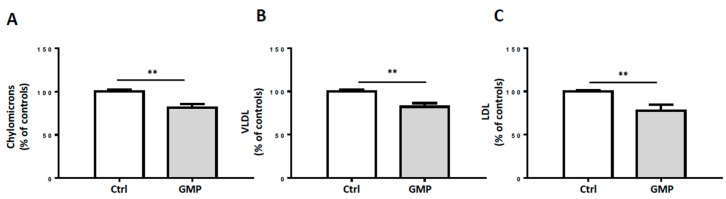
Effects of GMP on lipoprotein production in Caco-2/15 cells. Caco-2/15 cells were pre-incubated for 18 h with serum-free Eagle’s minimal essential medium (EMEM) containing BSA/oleic acid +/− GMP (2 mg/mL). After 18 h, medium was discarded and replaced with fresh EMEM and radiolabeled [^14^C]-oleic acid +/− GMP (2 mg/mL). After 24 h incubation, apical and basolateral media were collected. Lipoprotein production was assessed by quantification of (**A**) chylomicrons, (**B**) very low-density lipoproteins (VLDL), and (**C**) low-density lipoproteins (LDL) in basolateral medium. Results represent the means ± SEM of *n* = 3 independent experiments in triplicate. ** *p* < 0.01 vs. control (Ctrl) cells.

We further investigated the impact of GMP on lipoprotein production. Incubation of Caco-2/15 cells with oleic acid increased lipoprotein production of chylomicrons, very low-density lipoprotein (VLDL) and low-density lipoprotein (LDL) whereas the addition of GMP resulted in a reduced secretion of these lipoproteins (Figure 6).

## 4. Discussion

There is currently a growing interest in the use of dietary supplements and natural compounds to treat OxS and inflammation, two major processes contributing to the etiopathogenesis of many human chronic diseases. Although the health benefits of milk protein compounds are highlighted in many scientific reports, their impact on the gastrointestinal tract has not been investigated even if this system is central in cardiometabolic health homeostasis given its functional, physical, chemical, immunological, and microbiological properties. It is in this context that the present investigation has been undertaken. We could demonstrate that GMP, a milk-protein-derived peptide, is endowed with the potential to fight OxS, inflammation, intestinal lipoprotein production, and alterations of the insulin signaling pathways leading to postprandial dyslipidemia.

To examine the impact of GMP, we employed the Caco-2/15 cell line that undergoes a process of spontaneous differentiation leading to the formation of a monolayer of cells expressing several morphological and functional characteristics of the mature enterocyte [47,48]. As reported by our previous work, this remarkable intestinal model is regarded as the most appropriate for the investigation of gut absorption and interactions, nutrition, toxicology, food microbiology, bioavailability tests, and screening of drug permeability in discovery programs. Multiple studies from our laboratory [2,31,35,43,49,50] and from other groups have shown that Caco-2/15 monolayers are fully appropriate for the study not only of lipid/lipoprotein homeostasis [30,31,42,43,44], but also OxS and inflammation [35,40,41,51,52]. In addition to the intestinal model, we used the efficient Fe/Asc complex that displays great ability to induce lipid peroxidation [37,38,39,40,51,53]. In fact, iron initiates strong lipid peroxidation via the Fenton reaction whereas ascorbic acid amplifies iron’s oxidative potential by promoting metal ion-induced lipid peroxidation [51]. According to our previous studies, Fe/Asc not only generates reactive oxygen species (ROS), which strongly affect the prooxidant/antioxidant balance of the cell [37,38,39,40,51] but it also elicits inflammation [40,51]. Therefore, our experimental conditions are appropriate to evaluate the antioxidant and anti-inflammatory properties of GMP. Nevertheless, as a prerequisite to the present study, it was necessary for us to demonstrate that Caco-2/15 cells maintain their full integrity following Fe/Asc and GMP treatments. In line with our expectations, Fe/Asc and GMP did not exert cytotoxic effects on intestinal Caco-2/15 cell wholeness as noted by an in-depth analysis of cell viability, barrier integrity, and differentiation.

As anticipated, treatment with Fe/Asc led to a significant increase in MDA, a major biomarker of lipid peroxidation, and to a marked decrease in SOD2 and GPx, two powerful antioxidant enzymes, which indicates perturbations of the prooxidant/antioxidant balance. On the other hand, GMP prevented lipid peroxidation probably by keeping the endogenous antioxidants from crumbling. To approach the mechanisms of action triggered by GMP, we determined the protein level of the transcription factor NRF2, orchestrating the cellular defense against OxS by controlling the expression of antioxidant genes and conferring cytoprotection against OxS [40,41,54,55]. Under basal conditions, NRF2 is in its inactive form since it is associated with kelch-like ECH-associated protein 1 (Keap1), which convenes it to proteasomal degradation [56]. However, in response to cellular stimuli, NRF2 dissociates from Keap1 and translocates to the nucleus [57] where it binds to the antioxidant response elements (ARE) in the promoter region of antioxidant genes, thereby leading to their upregulation [58]. Our data clearly evidence the effectiveness of GMP to avert Fe/Asc-mediated NRF2 diminution, which explains the recovery of antioxidant enzymes. Our findings are consistent with two previous studies reporting the attenuation of oxidative damage via NRF2 improvement in HEPG2 cells and RAW 264.7 macrophages with GMP hydrolysates [59,60]. NRF2 capacity to prevent OxS is well known, but more recently, it has also been suggested to play a protective role against CMD [55]. Notably, pharmacological activation of NRF2 in obese mice on high-fat high fructose diet reversed IR and suppressed hepatic steatosis [61]. Furthermore, administration of a synthetic activator of NRF2 (triterpenoid CDDO-Imidazolide) to high-fat fed mice led to decreased body weight, adipose masse and hepatic lipid accumulation [62] Therefore, GMP mediated activation of NRF2 might indicate a potential application in the treatment of CMD.

Interestingly, GMP was also effective in depressing TNF-α and COX-2, two pro-inflammatory biomarkers in intestinal Caco-2/15 cells. As well evidenced by our findings, the mechanism for this GMP anti-inflammatory action is through the modulation of NF-κB/IκB pathway. In fact, GMP inhibited Fe/Asc-mediated NF-κB induction, which led to lower NF-κB/IκB ratio. Therefore, GMP may be an important peptide to guard against inflammation that endangers intestinal cell functions by controlling NF-κB, the master regulator of inflammatory response. Noteworthily, the upregulation of NRF2 may also contribute to the cellular defense against inflammation since dysregulation of NRF2 correlates with the development of chronic inflammatory diseases [63,64,65,66]. It is equally important to mention the crosstalk between OxS and inflammation processes [67,68]. For example, ROS can stimulate several inflammatory pathways through kinase activation and phosphorylation of NF-κB components [67,68]. On the other hand, NF-κB activation prevents NRF2 transcription by different mechanisms of action. In hepatocytes, it has been shown that overexpression of NF-κB p65 unit downregulates NRF2 transcription by competing with the same transcriptional co-activator CREB-binding protein (CBP)-p300 complex [69,70]. CBP-p300 plays a crucial role in histone acetylation, facilitating DNA transcription of histone, but also non histone proteins like NRF2 and p65 [69]. CBP-p300 has been shown to enhance the transcription of these target genes by adding an acetyl group on lysine residues of both transcription factors [70]. However, as CBP prioritizes NF-κB binding, it limits its availability for NRF2 [69]. Moreover, p65 may limit NRF2 transcription by promoting the recruitment of histone deacetylase 3, which associates with CBP [69]. Deacetylation of CBP prevents it from playing its co-activator role in NRF2, decreasing ARE-related gene expression [69]. Another proposed mechanism obtained from a hepatic cell line consists in p65 capacity to increase Keap1 levels [71]. This results in increase nuclear translocation of Keap1 and subsequent inactivation of NRF2. Those findings highlight the multiple pathways by which both OxS and inflammation influence each other. Studies are still required to explore how GMP targets these OxS-inflammation close interactions. As OxS and inflammation are key inducers of IR and other metabolic affections of the Metabolic Syndrome (MetS) [72,73,74,75], we decided to evaluate GMP’s capacity to regulate some key proteins of the insulin signaling pathway. We first focused on p38 MAPK that is activated by OxS and interferes with the insulin receptor signaling cascade [76,77]. Measurement of p38-MAPK phosphorylation and analysis of phospho-p38-MAPK/p38-MAPK ratio revealed a significant increase, which is compatible with reduced IS in Caco-2/15 cells. However, GMP displayed the ability to restore the values of p38 MAPK phosphorylation and phospho-p38 MAPK/p38 MAPK ratio to normal.

Thereafter, we turned to the PI3K/AKT signaling pathway that normally mediates the major biological effects of insulin by regulating glucose transport, protein synthesis, gluconeogenesis, cellular proliferation, and cell survival [78,79]. Instead, its abnormality directly results in IR [80]. Surprisingly, our experiments revealed a stimulation of the phosphorylation of PI3K/AKT by Fe/Asc, which returned back to normal with GMP administration. The impact of Fe/Asc-mediated lipid peroxidation on PI3K/AKT signaling is hitherto unknown in intestinal cells and comparisons are virtually impossible. A few studies have attempted to explain the mechanism of action by which OxS stimulates AKT signaling pathway. In DT40 cells, H_2_O_2_ was found to promote PI3K membrane recruitment to its substrate site, increasing PI3K catalytic function and AKT activation [81]. In glioblastoma cell line, H_2_O_2_ increases AKT phosphorylation via the upstream activation of focal adhesion kinase, a protein kinase involved in cellular adhesion which associates with PI3K [82]. In Hela cells, AKT activation is dependent of H_2_O_2_ stimulation of epidermal growth factor receptor [83]. Those different mechanisms of action highlight the numerous ways by which OxS can stimulate AKT, which are probably cell specific. However, more studies are needed in order to determine the exact mechanism of action by which Fe/Asc stimulates AKT in intestinal cells.

The explanation for our data appears to rest on the fact that AKT is involved in multiple signaling pathways other than insulin signaling such as cell survival, proliferation, metabolism, growth, and others [84]. AKT is particularly important for cell survival as it mediates important anti-apoptotic functions, limiting cell death induced by growth factor withdrawal, cell cycle discordance, and detachment of cells from their extracellular matrix [85]. However, conflicting data exist concerning the influence of OxS on AKT. Several studies have shown that OxS induced by pesticides in Caco-2 cells [86], UV irradiation (ASTC1 cells) [87], and hyperosmotic stress (kidney cells) [88] led to decreased AKT activity. However, other studies have shown that OxS induced by oxidant injury or other environmental stresses activate AKT [89,90,91]. For example, rat intestinal epithelial cells submitted to H_2_O_2_ results in increased AKT phosphorylation [92]. Similarly, H_2_O_2_ administration to hepatocytes induces ROS accumulation and increases AKT activity [93]. This discrepancy in AKT regulation seems to originate from the identity, intensity and persistence of the stress signal and the resulting fate of the cell. In studies where OxS was found to decrease AKT phosphorylation, cell apoptosis was also reported [86,87]. In contrast, in studies where OxS increased AKT phosphorylation, cell survival was instead promoted [82,83,92]. From those observations, several authors have put forward the idea that AKT downregulation is essential to the apoptotic process [85]. Since we demonstrated that Fe/Asc does not curtail cell viability (Figure 1), the enhanced AKT phosphorylation found in our investigation might indicate that Fe/Asc stimulates defense mechanisms against OxS and apoptosis in intestinal cells, which are mediated by AKT signaling pathway. Nevertheless, in most of the cases, GMP showed a tendency to bring the values of Fe/Asc-mediated insulin signaling factors closer to the control levels.

Since Fe/Asc is a pro-oxidant similar to H_2_O_2_ which induces ROS accumulation and antioxidant depletion, we hypothesize that the increase in AKT phosphorylation found in our results is indicative of a mechanism of resistance to OxS rather than a mechanism of IS. AKT also promotes cell survival by preventing the release of cytochrome c from the mitochondria [94]. Therefore, the enhanced phosphorylation of AKT in response to Fe/Asc in our study may reflect increased cellular defense mechanisms rather than stimulation of IS, which is consistent with similar outcomes following OxS and inflammation in other cellular models as demonstrated above [92,93,95]. Therefore, lower phosphorylation levels of AKT observed with GMP treatment may indicate a positive role of GMP against OxS and inflammation. This is supported by a previous study where GMP hydrolysates supplementation was also found to decrease AKT activity in lipopolysaccharides-stimulated RAW 264.7 macrophage [96]. Similar to our results, it was accompanied by a decrease in levels of malondialdehyde, ROS production and pro-inflammatory markers (NF-kB, COX-2, and phospho-p38-MAPK) [96]. Interestingly, the authors suggested that AKT phosphorylation could be involved in NF-kB activation, as it had been previously been demonstrated elsewhere [97,98]. Since we also found an increase in both AKT phosphorylation and NF-kB in our study, this mechanism of action is also a plausible signaling pathway. Although the literature is very sparse regarding the impact of iron on AKT signaling in intestinal cells, investigations conducted in other cell types can provide insight about its known effects. Studies conducted in neuroblastomas, hepatic macrophages, cerebral cortex synaptic endings and neurons, show that iron administration increases AKT activity in response to OxS [99,100,101,102]. Similar to our results, neurons exposed to iron display increased lipid peroxidation without alteration of cellular viability [103]. The authors reported that in response to OxS, AKT translocated to the nucleus and modulated glycogen synthase kinase 3β phosphorylation, Forkhead box O transcriptional activity, and glutathione metabolism that resulted in increased resistance to OxS [103]. Moreover, another study evidenced that iron deficiency downregulates AKT phosphorylation in rats and in COS-1 cells, leading to a decrease in mammalian target of rapamycin activity [104]. In line with this, in intestinal Caco 2 cells, iron depletion was also shown to depress AKT activity, whereas iron repletion restored AKT levels [105]. Therefore, it is reasonable to believe that the results obtained in our investigation might result from ROS accumulation induced by Fe treatment or by Fe activity itself.

We also assessed AMPK, a critical energy sensor and a well-known central regulator of energy metabolism. First, we noticed that the addition of insulin for the assessment of the insulin receptor signaling cascade resulted in decreased phosphorylation of AMPK, which has also been reported in diverse cellular models such as hepatocytes and hearts of rats [106,107]. Some authors suggested a direct inhibitory effect on AMPK, whereas others proposed an indirect impact [108,109,110]. In fact, different investigators insinuate that AMPK is phosphorylated on its inhibitory site (Ser 485/491) by AKT, which prevents its subsequent phosphorylation and activation on Th172 [111,112,113].

As GMP was observed to lessen OxS and inflammation, both known to induce lipid/lipoprotein disorders, we assessed the influence of GMP on lipoprotein output in Caco-2/15 cells. Our results revealed that GMP was effective in lowering the secretion of chylomicrons, VLDL, and LDL, suggesting its potency to prevent postprandial dyslipidemia and therefore CMD.

In summary, the present investigation demonstrates the capacity of GMP to improve IS and dyslipidemia, two major components of the MetS. GMP showed also beneficial effects on insulin signalization pathways and lipoprotein production. Therefore, GMP may represent a new preventive/therapeutic strategy to ameliorate metabolic disorders.

## Abbreviations

AMPK	AMP-activated protein kinase
ARE	Antioxidant response elements
CBP	CREB-binding protein
CM	Chylomicron
CMD	Cardiometabolic disorders
COX-2	Cyclooxygenase-2
EMEM	Eagle’s minimal essential medium
FBS	fetal bovine serum
Fe/Asc	Iron-ascorbate
GI	Gastrointestinal
GMP	Glycomacropeptide
GPx	Glutathione peroxidase 1
IκB	Inhibitor of kappa B
IS	Insulin sensitivity
IR	insulin resistance
Keap1	Kelch-like ECH-associated protein 1
LDL	Low-density lipoprotein
MDA	Malondialdehyde
MEM	minimal essential medium
MetS	Metabolic syndrome
MAPK	Mitogen activated protein kinase
NEAA	non-essential amino acid
NF-κB	Nuclear transcription factor-kappa B
NRF2	Nuclear factor erythroid 2–related factor 2
OxS	Oxidative stress
ROS	Reactive oxygen species
p-Akt	Phospho-protein kinase B
p38-MAPK	p38 mitogen activated protein kinase
SOD2	Superoxide dismutase 2
TNF-α	Tumor necrosis factor alpha
VLDL	Very-low-density lipoprotein
